# The Development of an Angiogenic Protein “Signature” in Ovarian Cancer Ascites as a Tool for Biologic and Prognostic Profiling

**DOI:** 10.1371/journal.pone.0156403

**Published:** 2016-06-03

**Authors:** Sofia-Paraskevi Trachana, Eleftherios Pilalis, Nikos G. Gavalas, Kimon Tzannis, Olga Papadodima, Michalis Liontos, Alexandros Rodolakis, Georgios Vlachos, Nikolaos Thomakos, Dimitrios Haidopoulos, Maria Lykka, Konstantinos Koutsoukos, Efthimios Kostouros, Evagelos Terpos, Aristotelis Chatziioannou, Meletios-Athanasios Dimopoulos, Aristotelis Bamias

**Affiliations:** 1 Department of Clinical Therapeutics, Medical School, National and Kapodistrian University of Athens, Alexandra General Hospital, Athens, Greece; 2 Metabolic Engineering and Bioinformatics Program Institute of Biology, Medicinal Chemistry & Biotechnology, National Hellenic Research Foundation, Athens, Greece; 3 First Department of Obstetrics and Gynecology, Medical School, National and Kapodistrian University of Athens, Alexandra General Hospital, Athens, Greece; Rajiv Gandhi Centre for Biotechnology, INDIA

## Abstract

Advanced ovarian cancer (AOC) is one of the leading lethal gynecological cancers in developed countries. Based on the important role of angiogenesis in ovarian cancer oncogenesis and expansion, we hypothesized that the development of an “angiogenic signature” might be helpful in prediction of prognosis and efficacy of anti-angiogenic therapies in this disease. Sixty-nine samples of ascitic fluid- 35 from platinum sensitive and 34 from platinum resistant patients managed with cytoreductive surgery and 1^st^-line carboplatin-based chemotherapy- were analyzed using the Proteome Profiler^TM^ Human Angiogenesis Array Kit, screening for the presence of 55 soluble angiogenesis-related factors. A protein profile based on the expression of a subset of 25 factors could accurately separate resistant from sensitive patients with a success rate of approximately 90%. The protein profile corresponding to the “sensitive” subset was associated with significantly longer PFS (8 [95% Confidence Interval {CI}: 8–9] vs. 20 months [95% CI: 15–28]; Hazard ratio {HR}: 8.3, p<0.001) and OS (20.5 months [95% CI: 13.5–30] vs. 74 months [95% CI: 36-not reached]; HR: 5.6 [95% CI: 2.8–11.2]; p<0.001). This prognostic performance was superior to that of stage, histology and residual disease after cytoreductive surgery and the levels of vascular endothelial growth factor (VEGF) in ascites. In conclusion, we developed an “angiogenic signature” for patients with AOC, which can be used, after appropriate validation, as a prognostic marker and a tool for selection for anti-angiogenic therapies.

## Introduction

Ovarian cancer is the most lethal gynecological type of cancer in developed countries. According to SEER data, approximately 23000 women will be diagnosed with ovarian cancer in the forthcoming years and about 15000 of them will die of the disease [1]. The lethality of this disease is mainly due to the fact that more than 75% of ovarian cancer sufferers present with advanced disease [2]. Treatment of advanced disease involves cytoreductive surgery combined with carboplatin/paclitaxel chemotherapy. Despite the initial effectiveness of this therapeutic approach, the majority of women will relapse, with a median PFS of around 18 months, and eventually die from ovarian cancer [3]. The one-size-fits-all approach does not account for the broad genomic and proteomic diversity of ovarian tumors. Accurate measurement of protein markers will be critical in distinguishing effective from ineffective therapies. An expanding pipeline of targeted therapies and increased appreciation for the molecular drivers within ovarian cancers have spawned a number of novel approaches for detection and treatment monitoring; these approaches include primarily blood tests for circulating tumor cells, tumor-derived exosomes, stem/progenitor cells, soluble tumor markers, as well as the use of genomic or proteomic information [4–8]. Nevertheless, there are still no reliable biomarkers capable of identifying ovarian cancer treatment failures before radiographic or biochemical evidence of progression.

Angiogenesis, is a process of production of new blood vessels and is a hallmark of cancer related to tumor survival and induction of tumor metastasis [9]. It constitutes of a dynamic process in which both pro-angiogenic and anti-angiogenic proteins are involved in the regulation of angiogenesis. Angiogenesis plays a major role in tumorigenesis, tumor expansion and ascites formation in ovarian cancer. The later represents an easily accessible biological fluid compared to tumor samples, while it may be more representative of the biological behavior of ovarian cancer compared with blood [10]. We have previously shown that VEGF levels are significantly higher in the ascites of women with advanced ovarian cancer compared to those in the serum of the same patients [11], suggesting that the angiogenic activity is most intense in the peritoneal cavity, the anatomical region of the highest disease burden. Moreover, high VEGF levels have been shown to be an adverse independent prognostic factor in advanced ovarian cancer patients being also associated with resistance to therapy [11]. Therefore, the inhibition of angiogenesis represents an important target in the fight against ovarian cancer. Currently, the anti-VEGF monoclonal antibody bevacizumab has been approved for primary treatment as well as treatment of relapse of ovarian cancer, while other anti-VEGF receptor tyrosine kinase inhibitors and anti-angiopoietin agents have shown efficacy in this disease [12]. Nevertheless, not all patients benefit from these therapies, which also have considerable toxicities.

For the above reasons, we hypothesized that an “angiogenic signature”, consisting of a panel of angiogenic factors that may be present in the patients’ ascitic fluid, might be an accurate prognostic tool as well as a means of selection of ovarian cancer patients likely to benefit from anti-angiogenic therapies. We hereby report the development of such “signature” based on the expression of 55 putative ovarian cancer markers of angiogenesis in ascites. In order to develop our model, we used resistance to chemotherapy as the discriminating factor to identify a favorable vs. an unfavorable angiogenic protein profile. The association of increased angiogenesis with chemoresistance is supported by a significant body of evidence from pre-clinical studies. Indicatively, pre-treatment of cancer cells with cisplatin induces a pro-angiogenic expression shift and leads to increased angiogenic activity when cells were treated with different chemotherapeutics including cisplatin [13]. In addition, cross-talk between VEGF and the anti-apoptotic bcl-2 protein has been described [14], while downregulation of bcl-2 leads both to reduced angiogenesis and sensitivity to chemotherapy and radiotherapy [15,16].

We found that a reduced marker set can form a promising tool, which is strongly associated with prognosis following cytoreductive surgery and standard cytotoxic chemotherapy. Following appropriate validation, this approach could be used for molecularly-directed anti-angiogenic therapy in ovarian cancer.

## Patients and Methods

### Patients

Sixty-nine patients suffering from ovarian cancer and fulfilling the following criteria participated in this observational, single-institution study: Stage III to IV (according to FIGO guidelines); patients should be scheduled to receive carboplatin-based 1^st^-line chemotherapy; patients had ascites at presentation; no anti-cancer treatment had been given before ascites collection. Ascites was prospectively collected but retrospectively analysed. All patients were treated at the 1^st^ Dept of Obstetrics and Gynaecology (University of Athens, Alexandra General Hospital, Athens, Greece) and the Dept of Clinical Therapeutics (University of Athens, Alexandra General Hospital, Athens, Greece). The study protocol had appropriate Institutional Review Board approval (Alexandra General Hospital, Athens, Greece) and written informed consent was given by all subjects for the collection and study of the ascitic fluid. The study was conducted according to the principles expressed in the declaration of Helsinki.

Ascitic fluid was collected prior to the administration of systemic therapy (either during primary cytoreductive surgery or paracentesis if laparotomy was not performed). It was mixed in sterile conditions with heparin (2u/ml) (LEO Pharmaceuticals, Denmark), centrifuged at 4°C at 1600 rpm, pellet was discarded and the supernatant was stored and cooled successively to -20°C for 1–2 hours and then transferred to of -80°C where it remained until used.

After treatment, all patients received follow up checks with CT scan every 6 months and CA-125 measurement every 3 months according to institutional protocol. Patients who relapsed during or within six months after completion of first-line chemotherapy were classified as platinum resistant, while patients exceeding 6 months without relapse were classified as platinum sensitive according to GCIG classification [17]. Relapse was determined using radiological or CA-125 progression (whichever was first) [17].

### Array based detection of angiogenic factors

Array detection was performed in 69 samples of ascitic fluid. In order to develop a prognostic “signature”, platinum resistance was used as a surrogate of prognosis: 35 samples from platinum sensitive patients and 34 samples from platinum resistant patients were analysed.

**Fig 1:**
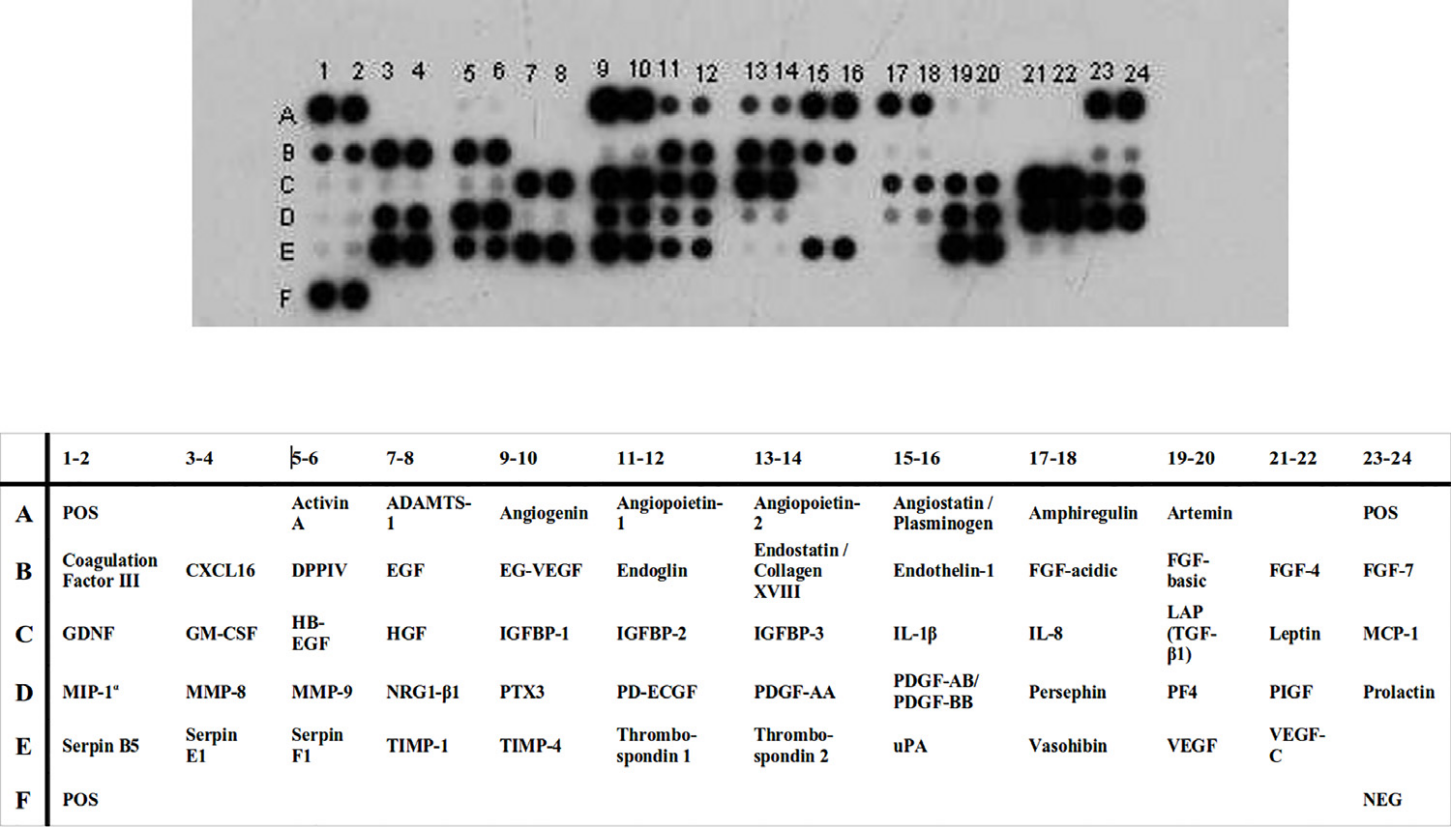
Angiogenic profile of patient with ovarian cancer. Demonstration of the angiogenic profile determined in the ascites of a patient with ovarian cancer using the Proteome Profiler Angiogenesis Array kit. Each spot corresponds to an angiogenic factor shown in Table 1.

The Proteome Profiler^TM^ Human Angiogenesis Array Kit, (R&D Systems, USA, Catalog Number: ARRY 007) [18] was employed to screen for the presence of 55 soluble angiogenesis-related factors **(Table 1)** in the patients’ ascitic fluid according to the manufacturer’s instructions. Briefly, 1ml of the supernatant from ascitic fluid is incubated with 500 μl of a relevant buffer and 15 μl of a mixture of biotinylated antibodies (specific for each of the 55 factors) for 1 hour. The mixture of ascitic fluid supernatant-antibodies-buffer is incubated for 16 hours at 4°C with a cellulose membrane coated with the same antibodies. Non-specific binding of antibodies to proteins of interest is avoided by pre-incubation of the membrane with a blocking buffer for 1 hour. The membrane is then incubated with streptavidin-HRP for 30 minutes. Light development is achieved through incubation with an ECL chemical reagent (R&D Systems) for 5 min. The emission of light is detected by using a special photographic film which depicts the protein trace. Thus the angiogenic proteins present in ascitic fluid are detected **(Fig 1)**. Quantitation of the detected spots was performed via standard densitometry using the Quantity One Software 4.4.1 (Bio Rad Laboratories Inc, CA, USA).

**Table 1:** The 55 angiogenesis-related factors used to distinguish between platinum resistant and platinum sensitive patients.

Protein Name		
Activin A	FGF-7	PDGF-AB/PDGF-BB
ADAMTS-1	GDNF	Persephin
Angiogenin	GM-CSF	Platelet Factor 4 (PF4)
Angiopoietin-1	HB-EGF	PlGF
Angiopoietin-2	HGF	Prolactin
Angiostatin/Plasminogen	IGFBP-1	Serpin B5
Amphiregulin	IGFBP-2	Serpin E1
Artemin	IGFBP-3	Serpin F1
FGF-4	IL-1β	TIMP-1
Coagulation Factor III	IL-8	TIMP-4
CXCL16	LAP (TGF-β1)	Thrombospondin-1
DPPIV	Leptin	Thrombospondin-2
EGF	MCP-1	uPA
EG-VEGF	MIP-1α	Vasohibin
Endoglin	MMP-8	VEGF
Endostatin/Collagen XVIII	MMP-9	VEGF -C
Endothelin-1	NRG1-β1	PDGF-AA
FGF acidic	Pentraxin 3 (PTX3)	
FGF basic	PD-ECGF	

In order to confirm our array findings, we studied 6 of the factors included in the array in 20 samples using enzyme-linked immunosorbent assay (ELISA) according to the manufacturer's instructions (R&D Systems, Minneapolis, MN). We chose factors showing significant differences in their array results between the two groups. In order to maintain a balance between the two groups, 3 factors showing increased expression among sensitive and 3 with increased expression among resistant patients were used.

### Optimal factor subset selection

The expression levels of the 55 angiogenic factors were used for feature selection and classification between platinum positive/resistant patients. The expression values were normalized according to the array specifications. The selection of the subset of factors that can most effectively separate the two classes was performed by recursive feature elimination with embedded cross-validation (RFECV) and using a Support Vector Machines (SVM) classifier. In order to estimate the best parameters for the SVM classifier, a grid scan approach was used, which consisted in automatically testing different combinations of parameters with the aim to maximize the Area Under Curve (AUC) criterion. Finally, the best performance was reported for the SVM classifier with the following parameters: linear kernel, C 0.001, gamma 0.001. Additional filtering was applied to the final subset using the entropy-based filtering methodology of Information Gain (IG), which measures the expected reduction in entropy caused by partitioning the samples according to a given attribute [19,20]. Factors with IG > 0 were retained. The application of feature selection to the initial dataset resulted to a subset of 25 factors the expression levels of which could best separate the two classes of patients (see Results).

### Evaluation of classifiers’ predictive performances through cross validation

For the selection of the best classifier to train with the reduced dataset of 25 factors (see Results), a grid scan in the parameter space of different classification algorithms (Support Vector Machines, Random Forests, Naive Bayes and Linear Discriminant Analysis) combined with 4-fold cross validation was performed (see Results section), in order to optimize the classification parameters, using as a criterion the Area Under Curve (AUC) maximization. This step confirmed that the best classification algorithm for this dataset was the SVM classifier (linear kernel, C 0.001, gamma 0.001). In order to further evaluate the performance of all classifiers in predicting the correct class (positive/resistant) of an 'unseen' patient (that has not been included in the training) when trained in all available labeled samples, we used a Leave-One-Out Cross Validation (LOOCV) approach. In the LOOCV classification scheme, a classifier is trained with all patient samples, except one, which is consecutively submitted to class prediction using the trained classifier (trained model). The process is repeated for each patient sample and yields an estimation of the performance of the classification in regard to unknown patient samples.

All aforementioned feature selection and classification procedures were implemented in the Python scikit-learn package.

### HeatMap representation

For the HeatMap representation the normalized expression levels of the 25 angiogenic factors were first log2 transformed and the relative expressions were estimated according to the median expression of each factor. One way (only expression values) hierarchical clustering was performed in GeneARMADA (linkage method: Average, distance: Cosine).

### VEGF ascites levels

VEGF in ascites was measured by ELISA according to the manufacturer's instructions (R&D Systems, Minneapolis, MN) as previously reported [11].

### PFS and OS analysis

The STATA/SE 11.2 SE software was used for statistical analysis. OS and PFS were defined as the time interval between the start of 1^st^-line chemotherapy and date of death from any cause and relapse of the disease; patients not dead or without relapse were censored on the date of last contact. Survival distributions were estimated using the Kaplan-Meier method; log-rank tests for equality of survivor functions between treatment groups were used. Cox proportional hazards regression analysis (univariate and multivariate) was performed to assess the influence on OS and PFS of the protein profile (“sensitive” vs. “resistant”), VEGF (> median vs. ≤ median), histology (serous vs. non serous), age (>60 vs. ≤60) and residual disease after primary cytoreduction (≤ 1cm vs. > 1cm). Variables with p<0.1 in univariate analysis were entered into the multivariate models.

## Results

### Patients

From March 2009 to January 2013 samples of ascites from 69 patients (35 platinum sensitive and 34 platinum resistant) with ovarian cancer were obtained. Their baseline characteristics are shown in **Table 2**. In all cases, ascites was obtained at initial diagnosis. In four cases, histology was not determined because no tumor biopsy was obtained during laparoscopy and the diagnosis of adenocarcinoma was based on the cytology of peritoneal fluid. All patients underwent primary cytoreductive surgery. All patients received first-line chemotherapy following cytoreductive surgery. Sixty—five patients received combination chemotherapy with Carboplatin / Paclitaxel, while 4 received Carboplatin monotherapy.

**Table 2:** Baseline characteristics of the 69 patients with advanced ovarian cancer included in this study.

*Characteristics*	*N (%)*
**Total**	69 (100)
**Age (median, range)**	63,5 (40–80)
**Histology**	
*Serous*	58 (84)
*Endometroid*	1 (1,5)
*Mucinous*	1 (1,5)
*Clear cell*	3 (4)
*Poorly differentiated*	2 (3)
*Unspecified*	4 (6)
**Chemotherapy administered**	
*Carboplatin / paclitaxel*	65 (94)
*Carboplatin*	4 (6)
**Stage**	
*IIIb*	2 (3)
*IIIc*	67 (97)
**Grade**	
*Low grade*	2 (3)
*High grade*	67 (97)
**Residual Disease**	
*< = 1cm*	29 (42)
*> 1cm*	40 (58)

### Confirmation of array findings with ELISA

All protein array data for the 55 factors are included in S1 Table. The ascites levels of 6 factors (Angiopoietin-1, TIMP-4, IL-1b, Endothelin-1, IGFBP-2 and MIP-1a) with significant differences in the array expression between sensitive and resistant patients were measured in 20 cases (10 sensitive and 10 resistant) with ELISA (Raw data in S2 Table). A similar trend between the array and the ELISA results was observed in all factors studied (**Table 3**).

**Table 3:** ELISA results (mean levels) of 6 factors with significant difference of expression in protein array.

	Sensitive (n = 10)	Resistant (n = 10)	Group of increased expression in array
**ANG-1**	1680.64	1317.64	Sensitive
**TIMP-4**	702.63	1316.21	Resistant
**IL-1b**	8.28	3.94	Sensitive
**Endothelin-1**	3.52	7.41	Resistant
**IGFBP-2**	619.25	624.27	Resistant
**MIP-1a**	624.13	74.78	Sensitive

### Results of feature selection and classification

**Fig 2:**
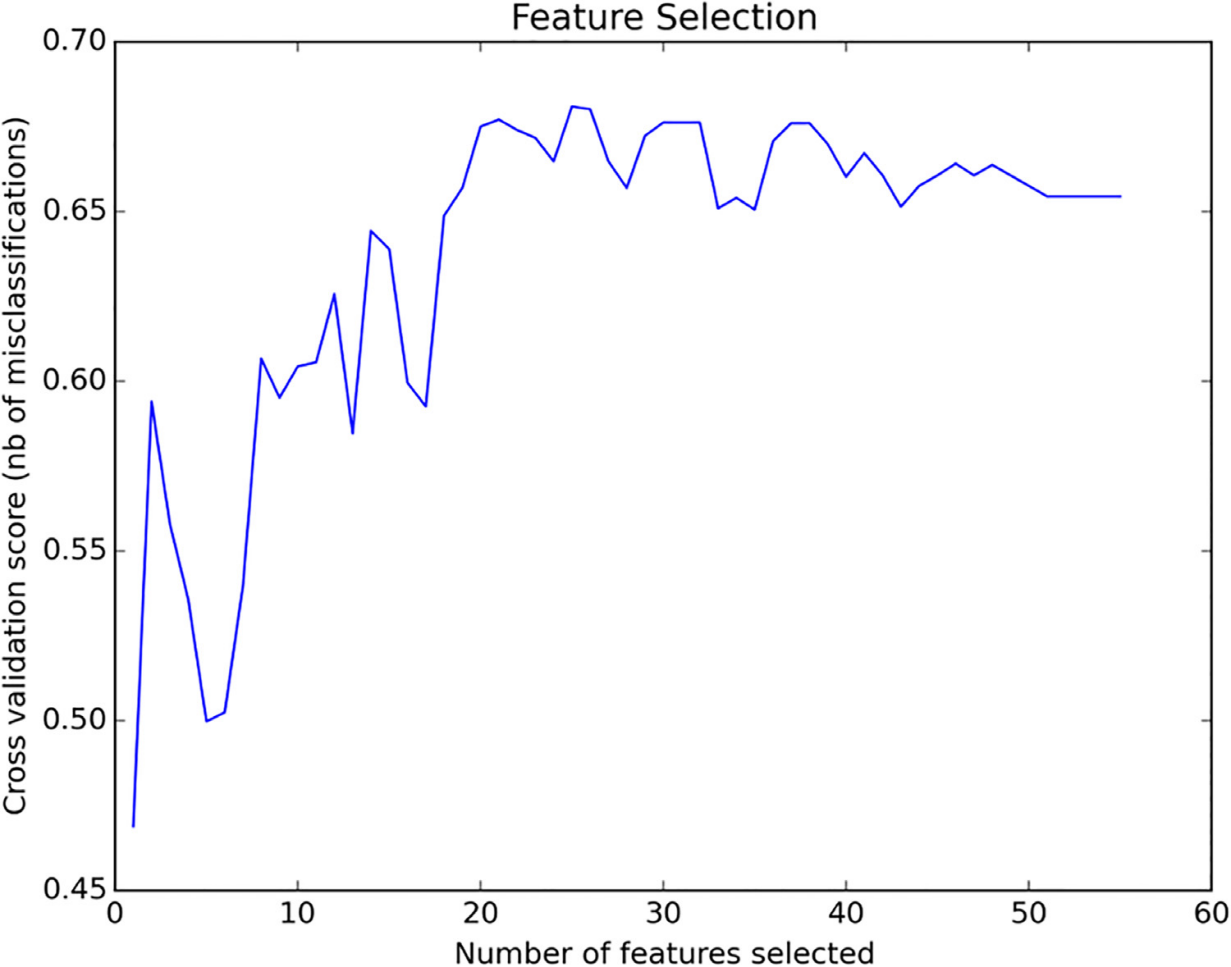
Feature selection cross validation scores. Feature selection cross validation scores, plotted against the number of selected factors. The optimal subset, maximizing the cross validation score, comprised 25 angiogenic factors (Table 3).

Despite the low dimensionality of the initial dataset (69 samples), an optimal combination of factors was found, the discriminative potential of which was evaluated with the utilization of four different classification algorithms. Feature selection was performed to find the optimal factor subset for correct classification, and resulted to a subset of 25 factors **(Table 4)**, which together maximized the 4-fold cross validation score **(Fig 2).**

**Table 4:** The subset of 25 factors included in the protein profiling and their corresponding pathways apart from angiogenesis.

	Factor	Pathways
1	Activin A	induction of expression of proteins such as FSH (follicle stimulating hormone) and epithelial to mesenchymal transition
2	Angiopoietin—1	activation of β1-integrin and N-cadherin promoting interaction between heamopoietic cells and the extracellular matrix in cancer
3	Angiopoietin—2	Signaling that leads to vascular permeability and it is also involved in septic shock
4	Amphiregulin	proliferation of epithelial cells via interaction with EGF and interacts with estradiol and progesterone for the development of mammary glands.
5	Coagulation Factor III	apoptosis signaling and thrombotic phenotype of cancer patients
6	EG—VEGF	proliferation of endothelial cells. Αcts as an autocrine mitogen for endothelial cells.
7	Endostatin / Collagen XVIII	Affects the Wnt signaling pathway that affects cell cycle progression
8	Endothelin—1	cell proliferation and apoptosis related pathways
9	FGF acidic	tumor cell proliferation and survival
10	FGF basic	Acts on proliferation of epithelial cells and in pathways affecting wound healing
11	HB—EGF	pathways that stimulate migration of cancer cells
12	HGF	pathways including PI3K, STAT3and cell proliferation
13	IGFBP—2	Through IGFR1 it is involved in cell proliferation, and through the IGFBP2/FAK pathway it is involved in chemoresistance
14	LAP (TGF - â1)	Accessory protein to TFG-b, may be involved through TGF-b in cancer cell progression
15	MCP—1	monocyte recruitment pathwaysand upregulation of cell survival
16	MIP - 1á	May increase osteoclast formation, and attracts machrophages and monocytes, involved in cancer cell proliferation
17	MMP-8	cell proliferation and migration pathways
18	PD-ECGF	MDR (Multi drug resistance) channels, attracts monocytes and leads to endothelial cell proliferation
19	PDGF-AA	Cell proliferation and survival
20	PDGF-AB/PDGF-BB	Cell proliferation and survival
21	Platelet Factor 4 (PF4)	Involved in cancer related thrombosis
22	PlGF	May enhance cell motility in cancer
23	Serpin F1	deterring cancer cell proliferation by inducing p53
24	TIMP- 4	cancer cell survival pathways
25	Thrombospondin—2	cancer cell proliferation and motility

**Fig 3:**
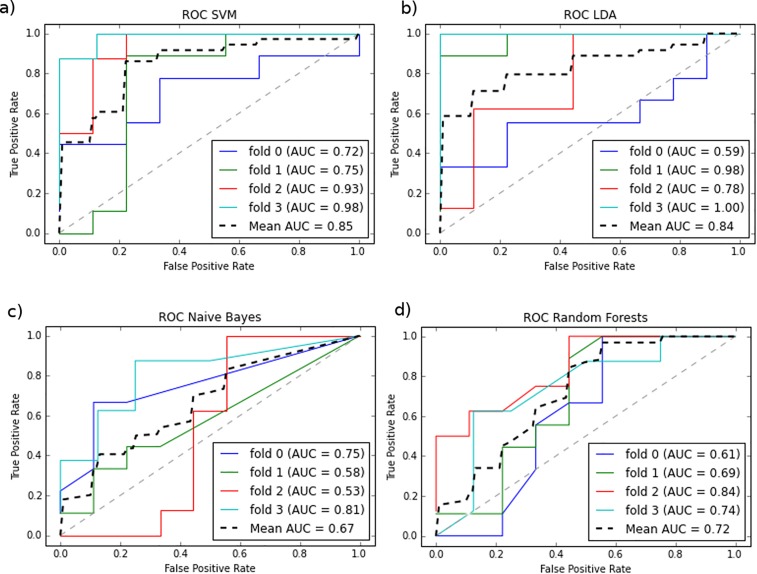
Classification algorithms. ROC curves, showing the performances of four different classification algorithms, applied to the reduced subset of four factors: a) Support Vector Machines b) LDA c) Naïve Bayes d) Random Forests. The SVM classifier optimally separated the positive and negative samples, with a mean AUC of 0.85. The other algorithms showed lower performances but still were able to classify the samples above the randomness cut-off of 0.50 AUC, and thus further confirmed the discriminative potential of the 25 factors.

The performances of the different classifiers, trained with the subset, are shown in (**Fig 3).** The SVM classifier had the maximum mean AUC (0.85). The other classifiers yielded lower scores but confirmed the discriminative potential of the factors subset, especially LDA with 0.84 mean AUC.

**Fig 4:**
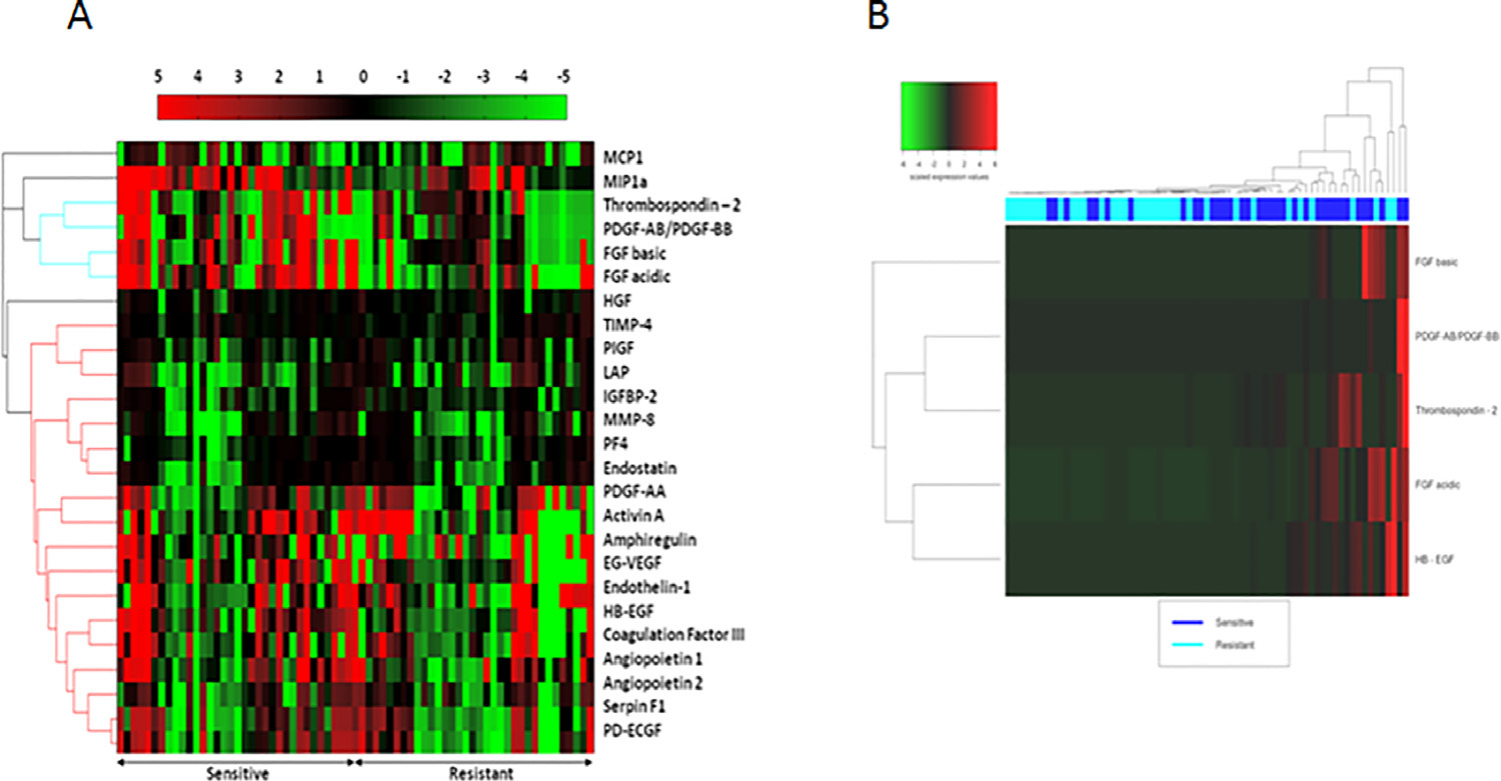
HeatMap of expression levels of the 25 angiogenic factors. Relative expression levels of the 25 angiogenic factors, which resulted in the maximum 4-fold cross-validation score (A) and the subset of 5 factors with the highest contribution to the signature (B). Expression values are displayed according to the colour scale, in which red represents above median expression and green represents below median expression. Given the complexity of the expression profiles, the two patient classes are not easily separated by clustering analysis, which justifies the utilization of more sensitive classification methodologies, like SVM.

The differences in the expression levels of these 25 factors among the patients are depicted in the HeatMap of (**Fig 4A**). For each patient, the expression level of each factor was compared to the median expression among all 69 patients. Since the visualization of the separation of the two groups was not ideal, we further filtered and ranked the 25 factors by applying an additional, entropy-based filtering method (information gain), which measures the importance of the factors with respect to patients classes using an entropy-based criteria. For the new heatmap representation we retained the 5 factors (FGF acidic, FGF basic, HB–EGF, PDGF-AB/PDGF-BB, Thrombospondin-2), that are identified by both Feature Selection methodologies and were ranked higher than the others in their contribution in the final signature. Hence the new heatmap with 5 factors depicts a subspace of the final signature which shows a tendency of over-expression of those factors in the Sensitive class and was more prone to visualization (**Fig 4B**).

The LOOCV method was used to further evaluate the predictive power of the classifiers trained with the 25 angiogenic factors' optimal subset as well as the subset of the 5 factors with the more satisfactory heatmap visulaization, in regard to effective classification of unknown patient samples (see Materials and Methods). By this approach the clinical value of the array kit as a diagnostic tool for sensitive/resistant patient classification is assessed more accurately, because LOOVC mitigates more effectively the phenomenon of “over-fitting” [21], in which the classifier’s model is excessively fitted to the training data and thus fails to correctly classify totally unseen samples.

The classifiers' performances are shown in **Table 5**. With the SVM classifier, 62 out of the 69 patients were classified correctly using the subset of 25 factors. This success rate (~ 90%) confirms that the angiogenic factor array kit can be used for accurate platinum sensitivity prediction in combination with the SVM algorithm. On the contrary, the subset of 5 factors resulted in low prediction performances.

**Table 5:** Performances of four different classification methods, combined with 4-fold and Leave-One-Out cross validation.

Classification Method	Mean AUC (4-fold CV)		Leave-One-Out CV correct predictions (%)	
	25 factors	5 factors	25 factors	5 factors
SVM	0.85	0.77	90	54
LDA	0.84	0.69	81	59
Naive Bayes	0.67	0.63	65	61
Random Forests	0.72	0.68	74	61

**AUC**: area under the curve; **SVM**: Support Vector Machines; **LDA**: Linear Discriminant Analysis; **CV**: Cross Validation

### Correlation of the angiogenic ‘signature’ with PFS and OS

For these analyses only the optimal “signature” resulting from the subset of the 25 factors was used. Median follow-up was 71 months (95% CI 48–99). During follow-up, 59 patients relapsed and 46 died from ovarian cancer. Median OS was 35 (95% CI: 24–41), while median PFS was 12 months (95% CI: 9.5–15). Histology, grade and residual disease after debulking surgery were not associated with PFS or OS. There was a trend towards shorter PFS and OS for women older than 60 years (p = 0.0832 and p = 0.0834, respectively).

**Fig 5:**
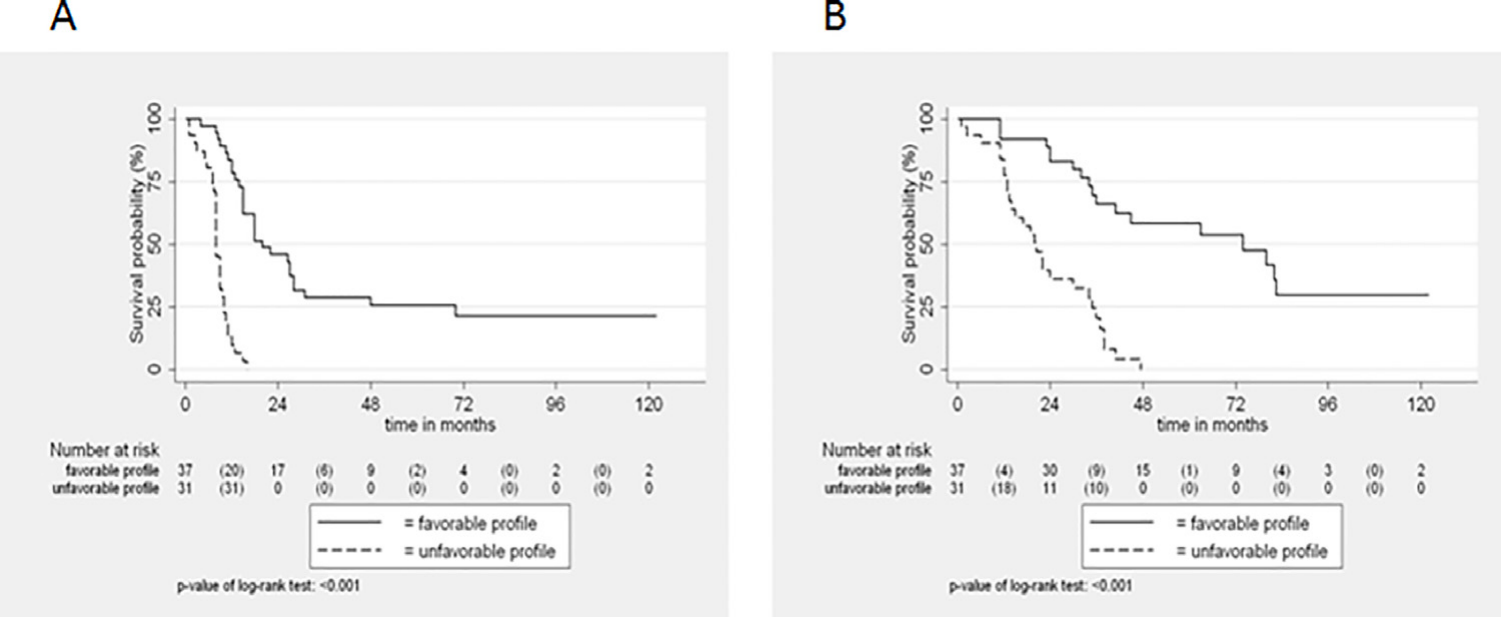
**Progression-free (A) and Overall (B) survival.** Progression-free (A) and Overall (B) survival of 69 patients with advanced ovarian cancer according to ascites angiogenesis-related protein profile.

Median VEGF ascites level was 1777 pg/ml. There was no association of VEGF level (below or above the median) with PFS (p = 0.836) or OS (p = 0.977). This result did not change when other cut off levels between 1000 and 2500 pg/ml were studied. There was no significant difference of the median VEGF level between the “resistant” and the “sensitive” protein profile (1680 vs 1779 pg/ml, p = 0.831). On the contrary, the “resistant signature” was associated with significantly inferior median PFS (8 months [95% CI: 8–9] vs. 20 months [95% CI: 15–28]; HR: 8.3, p<0.001) and OS (20.5 months [95% CI: 13.5–30] vs. 74 months [95% CI: 36-NR]; HR: 5.6 [95% CI: 2.8–11.2]; p<0.001) **(Fig 5)**. Multivariate analyses including age and protein profile showed that only protein profile retained its prognostic significance both for PFS (HR 7.9 [95% CI: 4–15.8], p<0.001) and OS (HR: 5.2 [95% CI: 2.6–10.5]; p<0.001).

## Discussion

In the current study, we developed and tested a bedside-compatible “angiogenic signature”, which was strongly associated with PFS and OS in women suffering from advanced ovarian cancer. The methodology we used has produced promising prognostic tools in other types of cancer [22–23], although different biological features were exploited. In line with our previous studies, we used ascites rather than serum. Ascites is a convenient source of biological material, which can be studied for diagnostic [23] and therapeutic purposes. In addition, certain proteins in ascitic fluid more accurately reflect the biology of the tumor than those in serum. The inclusion of pro- and anti-angiogenic factors is in concert to the in vivo mechanisms of the angiogenic activity, which is the result of the balance between these two groups in the microenviroment of the tumor. Angiogenesis is a valid surrogate for tumor behavior and panels of angiogenic factors present in tumor tissue have been recently selected for protein profiling of ovarian cancers [24]. In order to determine a “good” vs. a “bad” angiogenic profile we did not use an arbitrary time to relapse or death but platinum resistance instead. This surrogate has proven prognostic and predictive value [25] and provides a specific and validated time point for categorization of ovarian cancer patients.

Angiogenic profiling was based on the expression of 25 angiogenic factors (**Table 4**). Although VEGF plays a major role in the angiogenic process, it was not among them and VEGF levels in ascites did not predict for PFS or OS The latter could be attributed to the relatively small sample size but it should also be stressed that the prognostic and predictive value of this factor in ovarian cancer has been controversial. We and others have shown the prognostic significance of VEGF in serum or ascites in advanced ovarian cancer [11,26–28] but this factor has not been validated, while contradictory results do exist [29,30]. Regardless of the controversy surrounding the predictive and prognostic value of VEGF in ovarian cancer, our study showed that the prognostic value of the panel of the 25 factors was superior to that of VEGF alone, supporting the potential of our method. In spite of the lack of prognostic value of VEGF, several VEGF family members or factors interacting with VEGF were included in the panel. They are important signaling proteins involved in both vasculogenesis and angiogenesis, such as PIGF (placental growth factor), PD-ECGF (platelet derived endothelial cell growth factor, also known as thymidine phosphorylase [TP]) and PDGF-AB/PDGF-BB. Recent advances in our knowledge of neoangiogenic function have shown that these factors stimulate the full cascade of events required for angiogenesis and promote several potentially independent actions on the vascular endothelium such as endothelial mitogenesis, permeability, vascular tone, production of vasoactive molecules and the stimulation of monocyte chemotaxis [31]. Importantly, these factors have also been associated with resistance to chemotherapy, higher disease stage as well as prognosis in ovarian cancer [32–34]. Angiopoietin-1 (Ang-1) and Angiopoietin-2 (Ang-2) also seem to interact with VEGF to promote angiogenesis. They act via the Tie-2 receptor pathway and both have important roles in the molecular mechanisms of blood vessel formation. Ang-1 and Ang-2 seem to be targeted by paclitaxel chemotherapy [35]. Recently, targeting these factors has shown significant improvement of survival in recurrent ovarian cancer [36].

Several of the factors included in our “signature” have been correlated with response to current chemotherapy for ovarian cancer. Endothelin-1 (ET-1), is a powerful mitogenic peptide produced by different tumors including ovarian cancer. ET-1 was found to be over expressed in patients who do not respond to chemotherapy. This is probably associated with the interference of ET-1 on cell functions such as proliferation, drug-induced apoptosis, invasiveness and epithelial-mesenchymal transition [37–38]. Similar results related to chemoresistance to paclitaxel have been described for the angiogenic factor HB-EGF (heparin–binding epidermal growth factor) [39]. Two other factors that correlate with resistance to paclitaxel through inhibition of paclitaxel-induced apoptosis are acidic and basic fibroblast growth factor (FGF-a and FGF-b) [40]. They both play important roles in carcinogenesis development and invasion of ovarian epithelial cancer.

Finally, two factors of the epidermal growth factor family (EGF) were also included among the 25 significant factors. Tanaka et al [41] have shown that members of the EGF family play a pivotal role in the aggressive behavior of the tumor in ovarian cancer. Amphiregulin, is a well-studied protein, which has been found to be associated with the ovarian function and apoptosis of epithelial cells in ovarian cancer. Recent studies support that this factor has a highly significant correlation to cisplatin resistance [42]. On the other hand, the cytokine MIP-1α, is acting via the MAPK pathway. It interacts with VEGF via the upregulation of the MAPK pathway, especially ERK. Tran et al [43] demonstrated that MIP-1α induced a T-cell response in patients with ovarian cancer which was associated with a favorable outcome. It is, therefore, possible that, in spite of their association with angiogenesis, some of the 25 factors may exert their prognostic effect through other mechanisms, such as enhancement of immune response against the tumor.

Technical issues, such as the addition of heparin in the collected ascites, are unlikely to have affected our results. Although theoretically the presence of heparin might interfere with HB-EGF or VEGF levels, the amount of heparin we used is much lower than that used by standard manufacturers for effective coagulation, while the isoforms present may not be binding the ligand with high affinity as shown in other studies [44]. Our array results showed distinct differences of the expression of this factor among the samples studied (Fig 3), even from the same category i.e. chemoresistant or chemosensitive, therefore not rendering the array towards one or the other category specifically. This finding in combination with the fact that the development of our model was based on the differences of the expression between resistant and sensitive tumors and not on the absolute value of each factor make the effect of heparin on our model negligible. In addition, we found that the heatmap representation, combined with hierarchical clustering, may not be appropriate for visualizing such a complex signature. By filtering and ranking the 25 factors, we reduced the subset to the 5 most influential factors, thus producing a more satisfactory visualization. Nevertheless, this subset was associated with inferior predictive performance. This result supports the necessity of the utilization of machine learning methodologies to build predictive models, such as SVMs, which are more sensitive and able to capture complex discriminative patterns that reside in non-linear combinatorial differences in expression levels between the two classes. This is achieved by a non-linear mapping of original training data into higher-dimensional data, in order to find an optimal separating hyperplane in the new dimensions [45,46].

There are certain limitations associated with this report. The number of patients included was relatively low. Furthermore, a commercially available kit was used because our intention was to leverage protein profiling for eventual and widespread use in everyday practice. It is possible that other factors might also be useful but this would complicate the practicability of this method. In spite of the limited number of patients, the results are robust, suggesting that this method may be used to develop a reliable tool for defining prognosis and make therapeutic decisions in advanced ovarian cancer. For this reason validation in independent datasets is underway. It is important to underline that no anti-angiogenic therapies were used in the current cohort. In order to assess the potential of this model as a means of selection for anti-angiogenic therapies, ideally evaluation in a randomized population should be sought [25]. Nevertheless, anti-angiogenic effect is one of the mechanisms of action of current chemotherapy for ovarian cancer [47]. Thus, our findings support the potential for anti-angiogenic therapy selection of the developed “signature”.

Molecular factors not included in our array, such as gp100, ERCC, CD44, CD147, c-met, IL-6, ALDH, CD117 and BMP2 have also been studied in the context of chemoresistance in ovarian cancer. [48–51]. Although these molecules represent potentially interesting therapeutic targets, at the moment these results have been contradictive and not prospectively validated in large patient cohorts. In contrast to angiogenesis, no agents blocking their function are yet available, which puts the anti-angiogenic approach in a more mature position for the development of tools for patient selection for the respective therapies.

In conclusion, the panel of 25 angiogenesis-related factors provides a readily available tool, which can improve outcome prediction beyond that provided by age, grade, histotype and quality of primary surgery in patients with stage III and IV ovarian cancer. We believe that these findings may lead to the development of a tool to be used for patients’ selection for anti-angiogenic therapies. Future work is necessary to validate this model in prospective trials including patients treated with modern anti-angiogenic therapies.

## Supporting Information

S1 File Individual array figures for all 69 patients studied. (RAR)

S1 Table Full array results for the 55 angiogenesis-related factors studied. (XLS)

S2 Table Ascites levels of 6 angiogenesis-related factors with significant differences between sensitive and resistant groups in the protein array. (DOC)
