# Supplementary material for: The Development of an Angiogenic Protein “Signature” in Ovarian Cancer Ascites as a Tool for Biologic and Prognostic Profiling
Source: PLoS One. 2016 Jun 3;11(6):e0156403. doi: 10.1371/journal.pone.0156403 (PMC4892506; doi:10.1371/journal.pone.0156403)
Supplement: S2 Table — (DOC) [file pone.0156403.s003.doc]

S2 Table. Ascites levels of 6 angiogenesis-related factors with significant differences between sensitive and resistant groups in the protein array

| **ids** | **GROUP** | **Ang-1 (pg/ml)** | **TIMP-4 (pg/ml)** | **IL-1β (pg/ml)** | **Endothelin-1 (pg/ml)** | **IGFBP-2 (pg/ml)** | **MIP-1α (pg/ml)** |
| --- | --- | --- | --- | --- | --- | --- | --- |
| S1 | Sensitive | 1041,150 | 543,385 | 2,151 | 1,716 | 647,9 | 21,505 |
| R27 | Resistant | 1179,650 | 494,744 | 1,797 | 2,183 | 329,2 | 7,355 |
| R28 | Resistant | 1893,150 | 466,525 | 2,988 | 1,020 | 371,2 | 77,916 |
| S2 | Sensitive | 1381,250 | 718,270 | 6,633 | 5,537 | 648 | 715,517 |
| R29 | Resistant | 1318,500 | 662,945 | 2,628 | 20,587 | 907,9 | 96,52 |
| R30 | Resistant | 1161,850 | 648,093 | 6,187 | 15,798 | 554,2 | 377,31 |
| S3 | Sensitive | 1116,900 | 669,629 | 11,830 | 1,615 | 610,6 | 2721,59 |
| R31 | Resistant | 1360,200 | 1007,887 | 2,565 | 17,378 | 781,8 | 20,613 |
| S4 | Sensitive | 1517,900 | 765,425 | 2,820 | 3,031 | 736,4 | 33,477 |
| R32 | Resistant | 1273,150 | 1136,729 | 2,114 | 6,159 | 454,7 | 49,054 |
| R33 | Resistant | 1162,700 | 899,095 | 1,416 | 2,062 | 408,2 | 9,725 |
| S5 | Sensitive | 1109,950 | 507,740 | 5,157 | 9,763 | 748,6 | 2052,025 |
| S6 | Sensitive | 2601,150 | 833,003 | 9,233 | 1,292 | 743,9 | 54,41 |
| S7 | Sensitive | 1168,750 | 466,154 | 6,245 | 3,736 | 590,6 | 458,478 |
| R34 | Resistant | 1719,600 | 1956,198 | 2,489 | 2,644 | 1226,2 | 17,613 |
| S8 | Sensitive | 1363,650 | 588,313 | 2,705 | 6,633 | 444,7 | 41,613 |
| R35 | Resistant | 910,700 | 5250,000 | 10,482 | 1,622 | 453,5 | 18,447 |
| S9 | Sensitive | 1023,750 | 902,065 | 6,661 | 1,196 | 332,9 | 67,526 |
| R36 | Resistant | 1196,950 | 639,924 | 6,806 | 4,675 | 755,8 | 73,256 |
| S10 | Sensitive | 4482,000 | 1032,393 | 29,423 | 0,765 | 688,9 | 75,194 |
